# Harmony Behind the Trumped-Shaped Vessel: the Essential Role of the Ductus Venosus in Fetal Medicine

**DOI:** 10.4274/balkanmedj.2017.1389

**Published:** 2018-03-15

**Authors:** Sifa Turan, Ozhan M. Turan

**Affiliations:** 1 Division of Maternal Fetal Medicine, Department of Obstetrics, Gynecology and Reproductive Sciences, University of Maryland, Baltimore, USA

**Keywords:** Ductus venous, Doppler, fetal growth restriction, velocity ratios, hydrops, first trimester

## Abstract

The ductus venosus is a fetal vessel that functions importantly in the transfer of oxygen-and nutrient-rich blood from the umbilical vein to vital organs. Its control under active regulation and its anatomy result in a flow-velocity profile that is typically forward throughout the cardiac cycle. This forward cardiac function reflects afterload, cardiac contractility, compliance, and vascular volume changes. Ductus venosus assessment gives valuable information under different fetal conditions. For example, during first trimester screening, an abnormal ductus venosus measurement changes the screening result. Assessment of ductus venosus in twin-to-twin transfusion syndrome is an essential element of staging. In fetal growth restriction, an abnormal waveform mandates imminent delivery. In this review, we will discuss the role of ductus venosus assessment and its role in antenatal management and outcome prediction in certain fetal conditions throughout pregnancy.

## EMBRYOLOGY AND ANATOMY OF THE DUCTUS VENOSUS

The venous system develops from three bilaterally symmetrical sets of veins in the embryo: the cardinal veins, the vitelline veins, and the left and right umbilical veins. Ductus venosus (DV) is a part of the umbilical veins and develops as a channel in the liver as the liver grows and impinges on the remaining left umbilical vein. This small, trumpet-shaped vessel allows blood to flow from the umbilical vein to the left side of the inferior vena cava (IVC), near the entrance to the heart. The left side of the IVC receives blood from the DV as well as the left and medial hepatic veins and further directs the blood flow in a vertical direction toward the foramen ovale (1). The DV plays an important role in the distribution of oxygen (O_2_)-rich blood from the umbilical vein into the heart. Previous studies have demonstrated that the high kinetic energy of the blood from the DV contributes to the maintenance of an upward direction in the left compartment of the IVC to the foramen ovale (2). The shape of the vessel is also one of the factors that determine high-velocity flow at that level.

## SHUNTING PHYSIOLOGY

Many animal studies have shown that 50% of the umbilical blood is shunted through the DV (3,4). In human studies, under normal circumstances, the DV diverts 25% of umbilical venous flow toward the right atrium in this high-velocity stream, whereas 55% and 20% reaches the left and right liver lobes, respectively (5). The blood that enters the hepatic circulation eventually reaches the heart after metabolized in the liver. The trumped-shaped vessel plays an important role in this redistribution. An increase in the size of the vessel will eventually increase the amount of blood that reaches the heart, consequently downregulating the amount that enters the hepatic circulation. Some of the clinical conditions in this review will elaborate upon the possible size differences in the DV. In the venous vestibulum, O_2_- and nutrient-rich blood from the DV joins with the IVC and the three hepatic veins. However, the shape of the DV accelerates speed of the O_2_- and nutrient-rich blood. Therefore, DV flow does not mix with the IVC or hepatic veins. Similarly, it does not mix with the blood that originates from the superior vena cava or coronary sinus in the right atrium and passes to the left atrium through the foramen ovale. It is known that blood with a higher nutritional content is directed to the left ventricle and foramen ovale, which is the second shunt in the fetal circulation that provides allotment between O_2_- rich and depleted blood. As a consequence of this physiological distribution, the left ventricular output supplies the myocardium and brain with O_2_- and nutrient-rich blood (6).

## ASSESSMENT OF THE DUCTUS VENOUS BY DOPPLER ULTRASOUND

**Figure 1:**
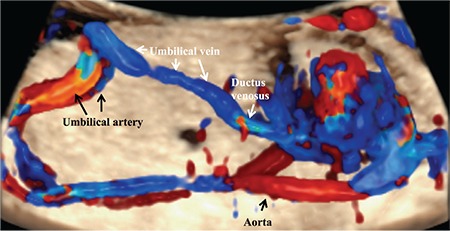
Sagittal view of the fetal chest and the abdomen using three-dimensional imaging with a glass body and color Doppler. The umbilical vein is continuous with the ductus venosus. The aliasing signal identifies the actual location of the ductus venosus.

**Figure 2:**
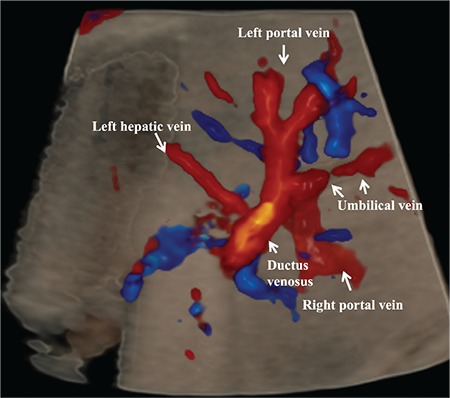
This figure shows a transverse view of the abdomen using three-dimensional imaging with a glass body and color Doppler. The aliasing signal identifies the actual location of the ductus venosus.

**Figure 3:**
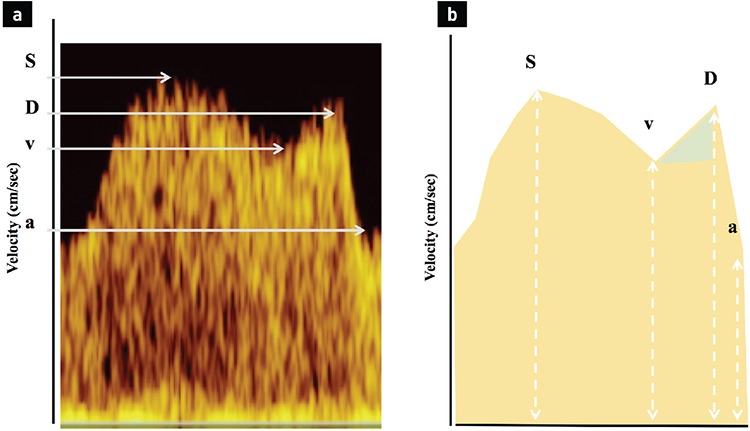
Normal ductus venosus wave form (a). The components of the multiphasic pattern: S (systole), D (diastole), v (v-descent), and a (a-wave) are marked with arrows. The Y axis demonstrates velocities. The measurements of velocities are depicted in (b). The green triangle represents the v/D ratio in a normal waveform.

The DV has a specific waveform. Here are some tips for obtaining the ideal waveform recording: recordings should be performed during fetal quiescence. Two anatomical positions of the fetus are ideal for recording. As a first option, color Doppler can be applied when the fetus is lying on its back; the mid-sagittal view through the fetal abdomen allows visualization of the DV connecting the umbilical vein to the IVC (Figure 1). The other option is the use of the transverse plane of the abdomen to visualize the DV. The probe should be aligned in the same direction as the blood flow through the DV (Figure 2). Color Doppler settings should be adjusted to identify high velocity vessel with alising: pulse repletion frequency = 2-3 kHz; or velocity limit = 30-40 cm.sec-^1^. The area of interest should be identified, and the color box must be adjusted with an appropriately magnified picture. Pulse wave Doppler settings should be applied with sweep settings of five waveforms, and the sample volume should be arranged to 2-5 mm and positioned over the isthmus and proximal section of the vessel (aliasing part). The high-pass filter should be adjusted to the lowest possible setting to prevent wall motion artifacts at the baseline; otherwise, identification of the reversed a-wave is not possible (7). Once the waveform is obtained, it can be used to assess pressure and volume changes in the heart during the cardiac cycle. The DV waveform consists of four phases, two of them seen as a peak and the other two seen as a trough. The S wave is the first peak in the waveform and represents systole in the cardiac cycle. This corresponds to the descent of the atrio-ventricular (AV) valve ring. At this time, the atrial pressure drops, venous return to the heart increases, and DV flow velocity rises. The second phase is v-descent (first trough) and represents ventricular relaxation during late systole. At this point the AV valve rings ascend toward resting position, atrial pressure rises, and DV flow velocity decreases. The third phase is the D wave (second peak), representing early ventricular diastole. At this time the AV valves open, atrial pressure decreases, and venous return increases a second time. Lastly, the “a”-wave represents atrial contraction during late diastole. Active ventricular diastolic filling occurs at this time, followed by an increase in atrial pressure (Figure 3a) (8,9).

## ANALYSIS OF THE DUCTUS VENOUS BY DOPPLER ULTRASOUND

It is very well known that potential changes in these velocities based on underlying pathology can be different and represent changes in cardiac status. These four phases in the waveform can be assessed in three different ways (Table 1). Firstly, the most common use in clinical practice is a semi-quantitative analysis of the pulstatility index for the vein (PIV). This measurement is angle independent and can be obtained by manual tracing through the S, D, v-descent, and “a” wave. The second option is qualitative (visual) assessment of, for example, a positive, absent, or reversed “a” wave (10). The third option is the assessment of velocity ratios, which could be a more comprehensive assessment of the cardiac cycle and can help us to better understand the cardiac physiology behind the pathological condition (Figure 3b) (11).

### Assessment of the Ductus Venous Doppler Waveform in the First Trimester

An association between Down syndrome and abnormal flow in the DV at 11-14 weeks has been found in several studies (12,13). Recent data show that abnormal flow in the DV can help improve the predictive capacity of increased nuchal translucency (NT) to detect major congenital heart defects (CHD) in chromosomally normal fetuses. Maiz et al. (14) found that in fetuses with increased NT and normal chromosomes following invasive testing, the finding of an absent or reversed a-wave in the DV is associated with a 3-fold increase in the likelihood of a major cardiac defect, whereas the finding of normal ductal flow is associated with a 50% reduction of the risk for such defects. Martínez et al. (15) reported that the detection rate of CHD improved from 28.9% to 40.0% in chromosomally normal fetuses with incorporation of increased NT and abnormal Doppler flow of DV in the first trimester. A recent meta-analysis reported that an abnormal DV a-wave could reveal 83% of CHD cases in the presence of an increased NT and 19% when the NT was normal, with a facilitated positional release (FPR) of 20% and 4%, respectively (16). A study of 40.000 singleton pregnancies with normal chromosomes at 11-14 weeks showed that screening for CHD using an increased NT and the reversed a-wave of the DV can detect 47.1% of major CHD with a FPR of 6.7% (17). Timmerman et al. (18) used the DVPIV instead of evaluating the DV flow patterns and found an abnormal DVPIV in two thirds of chromosomally normal fetuses with an increased NT and CHDs. The sensitivity of an abnormal DVPIV for CHDs was 70%, and the specificity was 62%. The DVPIV multiples of the median significantly influenced the risk of a CHD at any cut-off point, suggesting that the DVPIV can be used to increase the specificity of the NT in the identification of CHDs (18). Recently, Borrell et al. (19) assessed the best method to combine the NT and DV Doppler in the detection of major CHD in euploid fetuses and demonstrated different detection rates depending on the cut-offs of NT or DVPIV. They found that about half of all cases of major fetal cardiac defects would be detected by first trimester fetal echocardiography with the combination of NT and DV flow (19). The exact mechanism of the abnormal DV flow in the presence of CHD is still unclear, but abnormal DV flow may reflect impaired atrial contraction and reduced myocardial compliance with fluid accumulation behind the skin. Particularly, AV septal defects with AV valve regurgitation and hypoplastic left heart syndrome suggest that right atrial volume and pressure overload can reflect the DV and may be responsible for reversal of the DV a-wave and/or increased DVPIV. An abnormal DVPIV in the first trimester can detect approximately 70% of major CHD and is an indication for referral for specialized fetal echocardiography, even when the NT is normal (8). The other clinical indication for the use of DV Doppler in the first trimester is in early identification of Twin-to-Twin Transfusion syndrome (TTTS) in monochorionic twin pregnancies. Although there are other ultrasound parameters, such as nuchal translucency and the presence of tricuspid regurgitation along with abnormal DV Doppler in this group, the presence of an abnormality in DVPIV can be a sign of early manifestation of unbalanced shunting in these patients, which gives us a clue to follow the clinical progression of the diseases (20). In our clinical practice we suggest weekly follow-up ultrasound examinations when the DV is abnormal in a monochorionic twin pregnancy.

### Assessment of Ductus Venous Doppler Waveform in the Second and Third Trimester

There are several fetal conditions in the second and third trimester for which DV become very useful in clinical practice. The DV waveform reflects forward cardiac function by assessing compliance, contractility, and afterload of the heart. Management of these fetal conditions such as early onset fetal growth restriction (FGR), TTTS, non-immune hydrops, and structural and/or functional heart diseases require DV assessment.

## FETAL GROWTH RESTRICTION

**Figure 4:**
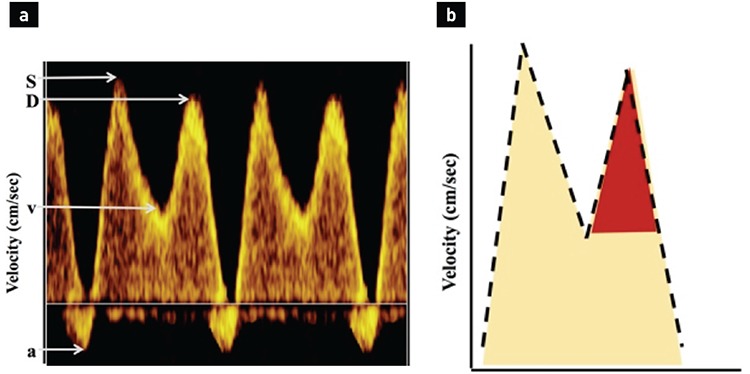
This is an “M”-shaped ductus venosus waveform, which is characteristic of increased afterload (a). A sharp decline in the v-descent and reversal of the a-wave are the characteristics of possible fetal hypoxia (Exp. fetal growth restriction, donor of twin-to-twin transfusion syndrome). The red triangle represents an abnormal v/D ratio (b).

It is widely accepted that there are two types of FGR; early onset and late onset. This classification not only describes the timing of the problem but also points to the etiology of the disease. DV changes and assessment are more critical in early FGR. The underlying pathogenesis in this group is impaired placentation due to abnormal trophoblastic spiral artery invasion. This begins during the second trimester and progresses in different patterns according to severity of the umbilical artery Doppler indices and the activation of fetal shunting mechanisms. Especially in severe hypoxia, extreme placental resistance leads to decompensation in the fetal heart, and venous Doppler changes appear. There are several plausible explanations behind the DV changes in early FGR, and these changes can be progressive. In the beginning of the process, increased shunting via DV occurs as a sign of divert shunting toward the heart instead of the hepatic circulation in order to save the main organs such as the brain and heart. This redistribution is reflected in the DV waveform in two different ways. Firstly, velocity during the S wave increases as a reflection of the increased blood flow through the vessel during systole. As the disease progresses, ventricular relaxation during late systole (v-descent) deepens. This process is typical for early FGR, because ventricular relaxation is an O_2_ dependent event. This early finding is not reflected in semi-quantitative analysis, for example, DVPIV values may not abnormal at the beginning of the disease. Ventricular relaxation is the major determinant at this point; therefore, only v-descent abnormalities will be visible if the S/v and v/D ratios are assessed. If the qualitative shape of the waveform is assessed, the “M”-shaped DV waveform represents v-descent abnormalities and is a waveform typical of FGR (11) (Figure 4a, 4b). In the event of severe hypoxia with lack of compensation, a-wave changes occur in the waveform. During atrial systole, venous forward flow decreases, reflected in the waveform as an absent or reverse a-wave. There is enough clinical evidence to accept that abnormal DV flow increases the risk of perinatal morbidity and mortality (21,22). An observational study showed that the time interval between the diagnosis of an absent/reversed a-wave and fetal death is approximately 5 days, irrespective of gestational age (22). In a recent randomized trial (the TRUFFLE study), the timing of delivery was determined based on three different antenatal assessment methods: reduced short-term variability, increased DVPIV and absent/reversed a-wave in DV. The study showed that delivery timing based on an absent/reversed a-wave was associated with an improvement in neurodevelopmental outcomes at 2 years of age (23). In our clinical practice, we suggest preparation for delivery if the DV a-wave is reversed or DVPIV is increased after 28-30 weeks. Aggressive fetal monitoring should be performed until delivery in both conditions.

In the late FGR, the pathology is quite different and is mainly driven by O_2_ and nutrient content, which have effects on hypoxemia. Therefore, abnormalities of cardiac function and DV abnormalities are not apparent in this group.

## TWIN-TO-TWIN TRANSFUSION SYNDROME

Unequal blood volume exchange between monochorionic twins (donor-recipient) can result in (TTTS). Arterial and venous Doppler changes have been used in the diagnosis and staging of TTTS. Cardiovascular manifestations can only be seen in later stages (stages 3 and 4) and differ between donor and recipient twins. Semi-quantitative or qualitative (visual) analysis of DV is useful to understand the disease severity as well as the timing of the intervention in this Complex syndrome. In donor twins, the main problem is reduced blood flow due to increased shunting toward the recipient; therefore, the DV waveform replicates the FGR fetuses. The v-descent is deeper and the a-wave is absent; visually, the “M”-shaped DV is the most common waveform seen in the donor, and this demonstrates abnormal ventricular relaxation. This effect can be determined by DVPIV measurement. When atrial contraction is affected by impaired ventricular filling, the a-wave appears absent or reversed, which can be diagnosed by visual assessment. The assessment of velocity ratios demonstrates abnormal S/v and v/D ratios (Figure 4b). On the other hand, in recipients the main problem is hypervolemia, myocardial hypertrophy, and high cardiac output, which potentially could progress to fetal hydrops. The effect of the above findings hemodynamically increases the preload and further impairs ventricular filling. As a consequence, the a-wave abnormalities in the DV become more prominent, and any semi-quantitative measurement or ratio that involves the a-wave will be abnormal in this group (24,25). It is clear from the published literature that DV abnormalities are more common in recipient twins than donor twins and have been included in the cardiovascular profiling score when the degree of cardiac manifestations are assessed in this group (26). The other benefit of using DV Doppler in TTTS is to monitor the response of the treatment after fetoscopic laser treatment. In the recipient, the DV waveform normalizes quickly if the treatment is successful. On the other hand, it is not surprising to see worsening of DVPIV in the donor twin due to acute volume changes as a sign of a sudden increase in the afterload. This transient change in the donor does not correlate with a poor outcome (27).

## FETAL HYDROPS

Venous Doppler has a value in non-immune fetal hydrops (NIFH). We would like to divide fetal hydrops into two different categories: cardiac disease-related  NIFH and non-cardiac disease-related  NIFH. The cardiac-related type would involve cardiac structure, function, and rhythm abnormalities.

## CARDIAC DISEASE-RELATED NON-IMMUNE FETAL HYDROPS

### Structural Cardiac Defects

The fetal circulation is designed as two parallel blood streams with differing nutritional content. This parallel nature enables fetuses with certain types of cardiac malformations to undergo normal fetal growth and development until term because systemic blood flow is adequate in utero and seldom leads to fetal hydrops. There are some structural cardiac malformations that can cause low-output hydrops, such as tricuspid dysplasia, Ebstein’s anomaly, pulmonary atresia with intact ventricular septum, and Absent Pulmonary Valve syndrome. The main problem in this group is altered forward cardiac function due to an increase in pressure on the right side of the heart, and to increased central venous pressure (preload) (28). Depending on the severity of the defect and cardiac compensation, which can differ from one fetus to another, DV Doppler can be measured as increased DVPIV with a present/absent or reversed a-wave. Serial assessment of DV Doppler and demonstration of progression of the worsening in indices or visual assessment in the Doppler waveform is usually more valuable and meaningful than a single measurement. Changes in DV waveform correlate with the risk of intrauterine or neonatal death, particularly in hydropic fetuses (29).

### Functional Cardiac Abnormalities

Cardiac arrhythmias, particularly supraventricular tachycardia (SVT) and cardiomyopathies, are classified as functional cardiac abnormalities. Inadequate diastolic filling is the main reason for abnormal cardiac function. At the beginning, SVTs can cause some immediate effects on the heart, such as valve regurgitation, decreased cardiac output, and increased central venous pressure. Persistence of SVT with the above findings will eventually lead to fetal hydrops. Cardiomyopathy develops as a late end result. DV Doppler indices will help us to not only identify the disease severity but also to observe effects of treatment and normalization of cardiac function. There is no doubt that DVPIV will be measured as abnormal in these cases, but when we examined the specific ratios S/v, S/D, v/D, and a-wave-related ratios, they were also abnormal compared with the multiphasic normal waveform (11). Treatment of arrhythmias with anti-arrhythmic medications can restore heart function. It is not unusual to demonstrate the normalization of the waveform visually by qualitative (visual) and semi-quantitative assessments, as well as with different DV ratios, prior to hydrops resolution.

## NON-CARDIAC DISEASE-RELATED NON-IMMUNE FETAL HYDROPS

Many Genetic syndromes, chromosomal defects, metabolic syndromes, and conditions such as sacrococcygeal teratoma, TTTS, Vein of Galen aneurysm, and placental tumors could lead high-output hydrops (30). DV indices in all aspects will indicate cardiac compromise and possibly the outcome. It is interesting that DVPIV abnormalities in high-output hydrops is rarely seen compared to low-output hydrops cases. Particularly in high-output hydrops, anemia is the first finding due to hyper-dynamic circulation as a compensatory mechanism to the anemia; increased cardiac output decreases the forward flow resistance in DV; therefore, significant pattern changes in the ratios or a severe reversal of the a-wave cannot be seen in this particular group. In overall abnormal venous flow in DV, an absent or reversed a-wave, along with umbilical vein pulsation is strongly associated with fetal demise in hydropic fetuses (31). In light of these Doppler indices and their etiology, appropriate treatment and management options for higher-risk fetuses should be evaluated and initiated.

In conclusion, fetal conditions that are affected by cardiovascular deterioration must be assessed with DV Doppler examination. This assessment not only assists in directing management but also helps to predict the perinatal outcome. Detailed evaluation of forward cardiac function using velocity ratios is used to clarify the underlying cardiovascular pathology.

## Figures and Tables

**Table 1:**
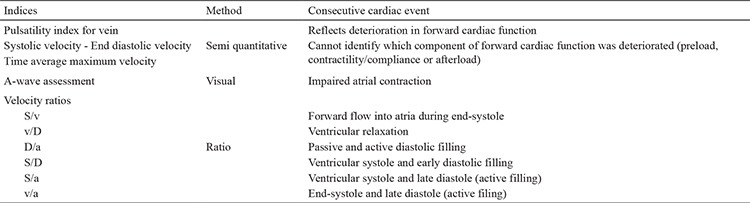
Ductus venosus wave form assessment methods
